# Risk Factors for Acute Kidney Injury in Coronary Artery Bypass Graft Surgery Patients Based on the Acute Kidney Injury Network Criteria

**Published:** 2018-04

**Authors:** Khosro Barkhordari, Ali Mohammad Fakhre Yasseri, Fardin Yousefshahi, Akbar Shafiee

**Affiliations:** 1 *Tehran Heart Center, Tehran University of Medical Sciences, Tehran, Iran.*; 2 *School of Medicine, Tehran University of Medical Sciences, Tehran, Iran.*; 3 *Imam Khomeini Hospital Complex, Tehran University of Medical Sciences, Tehran, Iran.*; 4 *Brain and Spinal Cord Injury Research Center, Neuroscience Institute, Tehran University of Medical Sciences, Tehran, Iran.*

**Keywords:** *Acute kidney Injury*, *Coronary artery bypass*, *Risk factors*

## Abstract

**Background:** Acute kidney injury (AKI) after coronary artery bypass graft surgery (CABG) is a common complication. The present study sought to determine AKI risk factors based on the Acute Kidney Injury Network (AKIN) classification.

**Methods:** In a cross-sectional study, performed from March 2010 to April 2012 at Tehran Heart Center, affiliated with Tehran University of Medical Sciences, 29 independent risk factors for AKI based on the AKIN criteria were examined in isolated post-CABG patients. The patients’ demographic data and risk factors were extracted from the Electronic Database of Tehran Heart Center. According to restricted inclusion and exclusion criteria as well as a creatinine rise to AKI Stage 1, the patients were divided into 2 groups of AKI-negative and AKI-positive and the risk factors were compared between these groups.

**Results:** Out of 3473 included patients at a mean age of 60.78 (±9.46) years, the majority (2474 [71.23%]) were male. Totally, 958 (27.7%) patients had AKI, according to a creatinine rise to AKI Stage 1. Logistic regression analysis demonstrated that higher age (OR=1.021; P<0.001), higher body mass index (OR=1.035; P<0.001), lower preoperative creatinine level (OR=0.417; P<0.001), longer cardiopulmonary bypass time (OR=1.004; P=0.007), blood transfusion in the ICU (OR=1.408; P=0.001), and lack of intraoperative blood transfusion (OR=0.823; P=0.044) were the independent risk factors for AKI after CABG.

**Conclusion:** Based on the findings of the current study, older age, higher body mass index, lower preoperative creatinine level, more blood transfusion in the intensive care unit (ICU), lack of intraoperative blood transfusion, and high cardiopulmonary bypass time may serve as risk factors for the development of AKI in CABG patients.

## Introduction

Acute kidney injury (AKI) after coronary artery bypass graft surgery (CABG) is a common event, causing a wide range of complications^1^ from longer hospital and intensive care unit (ICU) stays to increased risks of renal failure and hemodialysis and higher mortality rates.^2^^, ^^3^ AKI can, therefore, worsen the outcome of CABG and play a role in dependence on hemodialysis.^4^ On the other hand, CABG itself is usually performed in high-risk patients and is the main cause of AKI. With respect to CABG, operation duration and aortic cross-clamp time are some of the proven risk factors for AKI.^1^

The Acute Kidney Injury Network (AKIN) classification defines 3 stages for AKI. Stage 1 is classified if there has been a rise in the serum creatinine level of at least 0.3 mg/dL or 1.5- to 2-fold or if the urine output has decreased to less than 0.5 mL/kg/h in the last 6 hours. Stage 2 is classified if the serum creatinine level has increased by more than 2- to 3-fold or if the urine output has dropped to less than 0.5 mL/kg/h in the last 12 hours. And finally, Stage 3 is classified if the serum creatinine level has increased more than 3-fold or 4 mg/dL with an acute rise of 0.5 mg/dL or if the patient is on renal replacement therapy regardless of the prior stage or if the urine output has decreased to less than 0.3 mL/kg/h in the last 24 hours or if there has been anuria in the last 12 hours.^5^^, ^^6^

Previous studies have reported the incidence of 7.6% to 48.5% for AKI following CABG based on the AKIN classification. ^3^^, ^^6^^, ^^7^  Several risk factors for the development of AKI are mentioned in previous research.^1^^, ^^8^^-^^10^

The pathophysiology of AKI in the context of CABG can be divided into 3 categories: preoperative, intraoperative, and postoperative. The preoperative factors comprise heart failure, multiple ischemia, medications angiotensin-converting enzyme inhibitors, angiotensin receptor blockers, nonsteroidal anti-inflammatory drugs, contrast agents, antibiotics, vasopressors, vasoactive and other nephrotoxic drugs, anemia,^4^ and preoperative blood transfusion.^11^ The intraoperative factors are comprised of the hemodynamic effects of cardiopulmonary bypass (CPB) pump, cytokine and chemokine expression, inflammatory mechanisms, microscopic and macroscopic emboli, effects of aprotinin, and effects of the on-pump technique.^4^^, ^^12^^-^^14^ And finally, the postoperative factors consist of vasoactive drug use, contrast agents, hypovolemia, sepsis, postoperative heart failure,^4^ and re-exploration.^1^

A scoring system for the prediction of acute renal failure was suggested by Thakar et al.^8^ It is deserving of note, however, that the authors equated renal failure with dialysis after surgery.^8^

Studies on AKI based on the AKIN and RIFLE (Risk, Injury, Failure, Loss of kidney function, and End-stage kidney disease) classifications are mostly retrospective. In addition, the majority of these studies have used only the creatinine portion of the classification rather than the urine output.^15^ Furthermore, only a few studies have so far focused on the risk factors for AKI following CABG based on the AKIN classification and most of them have recruited inadequate sample sizes.^6^^, ^^16^

Currently, many physicians and investigators draw on the AKIN classification to discuss their patients’ condition. Accordingly, it is highly advisable that AKI risk factors be determined on the basis of the AKIN classification with a view to enhancing the patient selection criteria for CABG. We designed the present study to identify the risk factors for a rise in the serum creatinine level in the range of AKI Stage 1 in a large sample size of an Iranian population of CABG patients.

## Methods

Approved by the Research and Ethics Committee of Tehran Heart Center and the Research Committee of the Anesthesiology Department of Tehran University of Medical Sciences, the present cross-sectional study was performed from March 2010 to April 2012 at Tehran Heart Center, affiliated to Tehran University of Medical Sciences. In this study, 29 independent risk factors for AKI were examined in isolated post-CABG patients based on the AKIN criteria using the Cardiac Surgery Database of Tehran Heart Center.^17^ The inclusion criteria were composed of age above 18 years, elective surgery, availability of complete hospital records, ejection fraction greater than 30%, and preoperative serum creatinine less than 2 mg/dL. On the other hand, consumption of nephrotoxic drugs a week prior to surgery, history of preoperative hemodialysis, sepsis, and death in the first 24 hours after surgery were the exclusion criteria.

Totally, 7250 patients underwent CABG during the study period of approximately 2 years. A total of 3548 (48.9%) patients, who met the inclusion criteria, were recruited in the study and afterward 75 (2.1%) patients were excluded in accordance with our criteria. The final analysis was performed on 3473 patients.

The patients’ demographic, physiological, and clinical characteristics as well as their preoperative laboratory test and echocardiography results were extracted from the Electronic Database of Tehran Heart Center. This database includes past medical history variables such as heart failure, renal failure, dyslipidemia, hypertension, and smoking. These variables are labeled by research physicians on the basis of their accepted definitions by the hospital’s specialist committee on clinical and paraclinical criteria. For the purposes of the present study, data on intraoperative blood transfusion, number of operative transfusion units, ICU blood transfusion, CPB time (min), and aortic cross-clamp time (min) were obtained from the anesthesiologists’ reports in the operating room and the ICU.

If the time lapse between the last angiography and surgery was less than a week, previous angiography history was considered positive. AKI was measured according to the AKIN classification (only based on serum creatinine and not the urine output) in the current study. The definition of AKI in this study was a minimum of 0.3 mg/dL increase in the serum creatinine level or a 50% increase in baseline creatinine (rather than the urine output). Two serum creatinine levels were recorded in the database: the level obtained on the morning of the operation day and that obtained on the day after the operation. Some patients underwent surgery in the afternoon; as a result, the 2 measurements of the serum creatinine level were made in a period less than 24 hours. However, the difference between these values was considered AKI within 24 hours.

The patients were divided into 2 groups of AKI-negative (Group A) and AKI-positive (Group B), and the risk factors were compared between the 2 groups. 

The statistical analyses were performed using SPSS statistical software, version 19 (Chicago, IL, USA). Means ± standard deviations or medians with quartiles and frequencies with percentages were used to describe the continuous and categorical variables, respectively. The continuous variables were compared between the patients with and without AKI using the Student *t*-test. The categorical variables were compared between the mentioned groups using the χ^2^ test. Variables with p value less than 0.2 in the group comparison were selected to enter the multivariable model. A backward logistic regression model with the entry and removal probability of 0.1 was employed to determine the multiple predictors of AKI. A p value less than 0.05 was considered statistically significant for the predictors of AKI.          

## Results

With respect to the serum creatinine level only (without considering the urine output), from the 3473 patients included in the present study, 958 (27.7%) patients developed AKI Stage 1, while 62 (1.8%) developed AKI Stage 2 and 31 (0.9%) AKI Stage 3.

Our regression model for AKI Stage 2 and Stage 3 failed to work because of the proportionally low percentage of the cases, missing all the probable risk factors in the first steps.

Among the independent variables, age (P<0.001), body mass index (BMI) (P<0.001), ICU blood transfusion (P<0.001), CPB time (P=0.001), history of dyslipidemia (P=0.032), hypertension (P=0.040), and previous cardiac surgeries other than CABG (P<0.001) were significantly more frequent in Group B than in Group A.

Preoperative creatinine level (P<0.001) and height (P<0.001) were significantly higher in Group A. In Group B, the number of patients using insulin was significantly higher than that in Group A (P<0.001). Also in Group B, history of diabetes (P=0.268), chronic obstructive pulmonary disease (P=0.078), and cerebrovascular accident (P=0.185) was more frequent than that in Group A; the difference, however, did not constitute statistical significance. Furthermore, there was no significant difference between the 2 groups in terms of weight, gender, ejection fraction, smoking, renal failure, endocarditis, peripheral vascular disease, previous CABG, previous myocardial infarction, congestive heart failure, recent angiography (<1 wk), intraoperative blood units transfused, intraoperative blood transfusion, and clamp time. Comparisons of the baseline characteristics and operative-related variables are depicted in Table 1 and Table 2, respectively.

All the independent variables with a p value less than 0.2 were taken apart in our model of logistic regression analysis. The effect of each variable on AKI was tested statistically by this model. Among the independent variables, age (P<0.001), BMI (P<0.001), preoperative creatinine level (P<0.001), CPB time (P=0.007), ICU transfusion (P=0.001), and blood transfused in the operating room (P=0.044) had an effect on AKI risk. The odds ratio of each of these variables was also measured by this model. Table 3 illustrates these risk factors as well as their p value and odds ratio. Judging by the β coefficient, it can be concluded that AKI risk increased 0.021-fold with a 1-year increase in age, AKI risk increased 0.034-fold with a 1-kg/m^2^ increase in the BMI, AKI risk increased 0.875-fold with a 1-mg/dL decrease in the preoperative creatinine level, AKI risk increased 0.004-fold with a 1-minute increase in CPB time, AKI risk increased 0.342-fold with blood transfusion in the ICU, and finally AKI risk increased 195-fold with lack of blood transfusion in the operating room.

**Table 1:** Comparison of the independent variables between the patients with and without AKI*

	AKI negative (n=2515)	AKI positive (n=958)	P
Age (y)	60.35±9.51	61.92±9.13	<0.001
Male gender	1796 (71.4)	678 (70.8)	0.229
Weight (kg)	73.42±12.25	73.64±11.75	0.632
Height (cm)	165.20±9.84	163.77±9.78	<0.001
BMI (kg/m^2^)	26.84±4.03	27.47±4.24	<0.001
Diabetes mellitus	857 (34.1)	356 (37.2)	0.268
Diabetes control type			0.045
Oral	655 (81.4)	258 (75.2)	
Insulin	108 (13.4)	65 (18.2)	
Mixed	42 (5.2)	20 (5.6)	
Smoking			0.116
Current	360 (14.3)	126 (13.2)	
Former	518 (20.6)	180 (18.8)	
Dyslipidemia	1331 (52.9)	557 (58.1)	0.032
Hypertension	1243 (49.5)	519 (54.2)	0.040
Renal failure	6 (0.2)	2 (0.2)	0.970
COPD	36 (1.5)	24 (2.5)	0.078
CVA	83 (3.3)	41 (4.3)	0.185
Previous CABG	5 (0.2)	2 (0.2)	0.959
Previous MI	898 (35.8)	316 (33.0)	0.220
Congestive heart failure	49 (1.9)	20 (2.1)	0.106
Previous angiography (< 1 wk)	302 (12.0)	113 (11.8)	0.815
EF (%)	47.68±8.38	47.53±8.60	0.613
Preoperative creatinine (mg/dL)	0.96±0.35	0.89±0.38	< 0.001

AKI, Acute kidney injury; BMI, Body mass index; COPD, Chronic obstructive pulmonary disease; CVA, Cerebrovascular accident; CABG, Coronary artery bypass graft; EF, Ejection fraction; MI, Myocardial infarction

* Data are presented as mean±SD or n (%)

**Table 2:** Comparison of the intra- and postoperative variables between groups*

	AKI negative (n=2515)	AKI positive (n=958)	P
CPB time (min)	71.83±29.57	71.83±29.57	0.001
Clamp time (min)	43.69±15.02	43.69±15.02	0.838
Intraoperative blood transfusion	604 (24.2)	604 (24.0)	0.421
Intraoperative transfusion units	0.24±0.42	0.24±0.42	0.421
ICU blood transfusion	364 (14.5)	364 (14.5)	<0.001

AKI, Acute kidney injury; CPB, Cardiopulmonary bypass; ICU, Intensive care unit

*Data are presented as mean±SD or n (%)

**Table 3:** Results of the logistic regression analysis of the risk factors for the development of AKI in patients after CABG

	OR	95% CI	P
Age	1.021	1.012-1.030	<0.001
BMI	1.035	1.015-1.054	<0.001
Preoperative creatinine level	0.417	0.296-0.586	<0.001
CPB time	1.004	1.001-1.006	0.007
ICU blood transfusion	1.408	1.142-1.737	0.001
Intraoperative blood transfusion	0.823	0.681-0.995	0.044

AKI, Acute kidney injury; CABG, Coronary artery bypass graft; BMI, Body mass index; CPB, Cardiopulmonary bypass; ICU, Intensive care unit

## Discussion

In the present study, we found that that old age, high BMI, blood transfusion in the ICU, lack of blood transfusion in the operating room, long CPB time, and low preoperative creatinine level were the potential risk factors for AKI. The patients’ age and BMI were significantly different between our 2 study groups. This is the first study to suggest a low preoperative creatinine level as a risk factor for the development of AKI in CABG patients with a preoperative creatinine level of less than 2 mg/dL. This finding does not chime in with some other similar investigations.^14^^, ^^18^ The reason for this discordance with other studies may be found in the different inclusion criteria of our study insofar as we excluded patients with serum creatinine levels over 2 mg/dL. It can also be concluded that in patients with creatinine levels lower than 2 mg/dL, a drop in the level would lead to an increased risk of AKI. Since the serum creatinine level does not correlate with the glomerular filtration rate (GFR), this finding could be factitious and it could have been reversed had the GFR been calculated instead of the raw creatinine value. As our unproved hypothesis by authors, it could be the result of the exclusion of patients with higher creatinine levels as well as the application of some prophylactic considerations in patients with higher creatinine levels or more liberal application of potentially nephrotoxic agents in patients with lower creatinine levels. 

In the raw comparison, hyperlipidemia and hypertension were significantly different between the 2 groups in the current study; this result was in agreement with those of other studies.^19^ Nonetheless, this finding was not confirmed by our regression assay, which means that the effects of hyperlipidemia and hypertension on AKI are due to other comorbidities.

Whereas some studies have reported male gender as a risk factor for AKI, we found no statistically significant difference between our study groups apropos gender. Similar to all the previous studies but one by Parolari et al.,^18^ we found no significant relationship between smoking and AKI, and nor did we detect a significant relationship between redo CABG and AKI; this finding is in line with most of the previously conducted studies in this field. Only the studies by Karkouti et al.^1^ and Vellinga et al.^16^ mentioned re-operation as an AKI risk factor; nonetheless, neither of the 2 studies was based on the AKIN classification. The fact that there were only a few redo CABG cases in our study (only 7 patients) precluded us from conclusively demonstrating the absence of a correlation between re-operation and AKI. 

There was no correlation between AKI and recent angiography in our study population. This finding is concordant with the results of all the previous studies, with the exception of that by Kim et al.,^19^ who did not exclude preoperative renal failure. As a result, it can be concluded that the contrast agent might be a risk factor for AKI only in patients with previous renal impairment and that it may not affect patients with normal kidneys.

In contrast with other studies, there was no significant difference between our 2 groups of AKI-negative and AKI-positive patients with respect to congestive heart failure and the ejection fraction. The fact that an ejection fraction over 30% was one of our inclusion criteria led to the recruitment of patients with only mild heart failure, which is likely to be responsible for the nonexistence of a correlation between AKI and heart failure. 

We found no significant differences regarding weight, renal failure, endocarditis, peripheral vascular disease, previous valvular surgery, and previous myocardial infarction between our 2 study groups. 

In the present study, there was a relationship between AKI and CPB time but not with cross-clamp time, which does not tally with other studies.^1^ It can be concluded that patients with mild cardiac and renal impairment (as was the case with the patients in the present study) have more resistance to the ischemic effects of long aortic clamping as it is known in cardiac and renal preconditioning.^20^^-^^22^

There was no significant difference between our 2 groups of AKI-negative and AKI-positive patients in terms of preoperative blood transfusion and transfused units. Our study population did not have this condition prior to CABG because of the inclusion criteria, which may explain the divergence between the findings. 

Our results demonstrated a statistically significant relationship between AKI and ICU blood transfusion. This could be corresponding with the findings of previous studies which suggested that in critically ill patients with a pre-inflammatory state, blood transfusion might be a risk factor for AKI.^9^^, ^^11^^, ^^16^^, ^^18^ In contrast, intraoperative blood transfusion was associated with a decrease in the risk of AKI in our patients. This is a novel finding and could be due to enhancement in renal perfusion and prevention of the inflammatory effects of transfused blood by washout plasma or pro-inflammatory cytokines by CPB filtering systems. Specially designed studies are required to sufficiently assess this finding.

One of the strengths of the present study lies in its inclusion criteria. We tried to exclude preoperative renal hypoperfusion causes and compare real AKI between 2 groups of AKI-negative and AKI-positive patients. 

Firstly, only 2 creatinine levels (i.e., preoperative and postoperative measurements) were recorded from the patients in the database. Changes in creatinine levels after a renal insult may be a few days behind changes in renal function; we may, therefore, have missed some changes by measuring the creatinine level in the first 24 hours after surgery. Secondly, some of our patients underwent surgery in the afternoon; consequently, the AKI measurement in these patients was not exactly within 24 hours. Thirdly, the urine output was not recorded in the database, leading to underestimation of AKI incidence. Fourthly, we focused on a rise in the serum creatinine level within the range of AKI Stage 1 and since our database lacked data on several possible confounders such as the use of diuretics, vasopressors, and inotropes, we could not assay their effects on AKI incidence. And finally, due to our strict criteria, we might have underestimated the incidence of AKI in this study.

## Conclusion

Our results identified old age, high BMI, high CPB time, ICU blood transfusion, lack of intraoperative blood transfusion, and low preoperative creatinine levels as AKI risk factors in the candidates for CABG. Further studies are required to determine AKI risk factors post CABG based on AKI Stage 2 and Stage 3 and shed more light on the effects of intraoperative blood transfusion.

## Notes:

***This paper should be cited as: ***Barkhordari K, Fakhre Yasseri AM, Yousefshahi F, Shafiee A. Risk Factors for Acute Kidney Injury in Coronary Artery Bypass Graft Surgery Patients Based on the Acute Kidney Injury Network Criteria. J Teh Univ Heart Ctr 2018;13(2):52-57.
